# Synthesis, Characterization, and Biological Activity of New 4‘‐Functionalized Bis‐Terpyridine Ruthenium(II) Complexes: Anti‐Inflammatory Activity Advances

**DOI:** 10.1002/cmdc.202400680

**Published:** 2024-11-21

**Authors:** Mohamed M. Elnagar, Khaled S. Abou‐El‐Sherbini, Safia Samir, Walid Sharmoukh, Mohamed S. Abdel‐Aziz, Yasser M. Shaker

**Affiliations:** ^1^ Institute of Electrochemistry Ulm University Albert-Einstein-Allee 47 89081 Ulm Germany; ^2^ Department of Inorganic Chemistry National Research Centre 33 El Bohouth St. (former Tahrir St.) 12622 Dokki, Giza Egypt; ^3^ Department of Biochemistry and Molecular Biology Theodor Bilharz Research Institute P.O. Box 30 Giza Egypt; ^4^ Department of Microbial Chemistry National Research Centre 33 El Bohouth St. (former Tahrir St.) 12622 Dokki, Giza Egypt; ^5^ Division of Pharmaceutical and Drug Industries Department of the Chemistry of Natural and Microbial Products National Research Centre El Buhouth Street Dokki, Cairo 12622 Egypt

**Keywords:** Ru(II) complexes, Terpyridine, Biological activity, Antimicrobial agents, Anti-inflammatory, Anti-cancer

## Abstract

Ruthenium complexes incorporating 2,2′ : 6′,2′′‐terpyridine ligands have emerged as promising candidates due to their versatile biological activities including DNA‐binding, anti‐inflammatory, antimicrobial, and anticancer properties. In this study, three new 4′‐functionalized bis(terpyridine) Ru(II) complexes were synthesized. These complexes feature one ligand as 4‐(2,2′ : 6′,2′′‐terpyridine‐4′‐yl) benzoic acid and the second ligand as either 4′‐(2‐thienyl)‐2,2′ : 6′,2′′‐terpyridine, 4′‐(3,4‐dimethoxyphenyl)‐2,2′ : 6′,2′′‐terpyridine, or 4′‐(4‐dimethylaminophenyl)‐2,2′ : 6′,2′′‐terpyridine. Besides the chemical characterization by ^1^H and ^13^C NMR, mass spectrometry, and absorption and emission spectroscopy, the complexes were tested for their biological activity as anti‐inflammatory, anticancer, and antimicrobial agents. Moreover, the toxicity of the Ru(II) complexes was assessed and benchmarked against diclofenac potassium and ibuprofen using a haemolysis assay. Biological evaluations demonstrate that these ruthenium complexes exhibit promising therapeutic potential with reduced haemolytic activity compared to standard drugs. They demonstrate substantial anti‐inflammatory effects through inhibition of albumin denaturation along with moderate cytotoxicity against cancer cell lines and broad‐spectrum antimicrobial activity. These findings highlight the multifaceted biomedical applications of 4′‐functionalized bis(terpyridine) Ru(II) complexes, suggesting their potential for further development as effective and safe therapeutic agents.

## Graphical abstract

This study presents the synthesis and characterization of new 4′‐functionalized bis(terpyridine) ruthenium(II) complexes. Biological assessments reveal low hemolytic potential, effective anti‐inflammatory activity, selective cytotoxicity against cancer cells, and broad‐spectrum antimicrobial effects, indicating their suitability for further development as effective therapeutic agents.

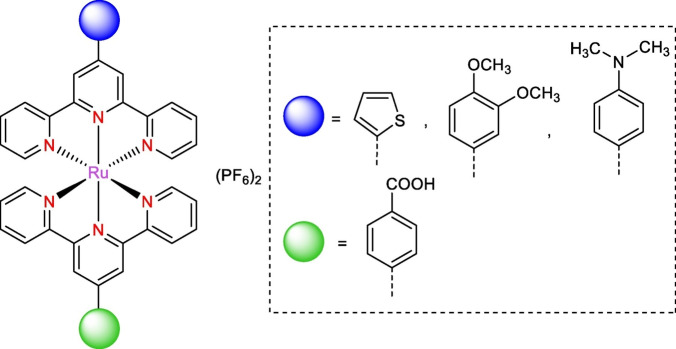

## Introduction

**Figure 1:**
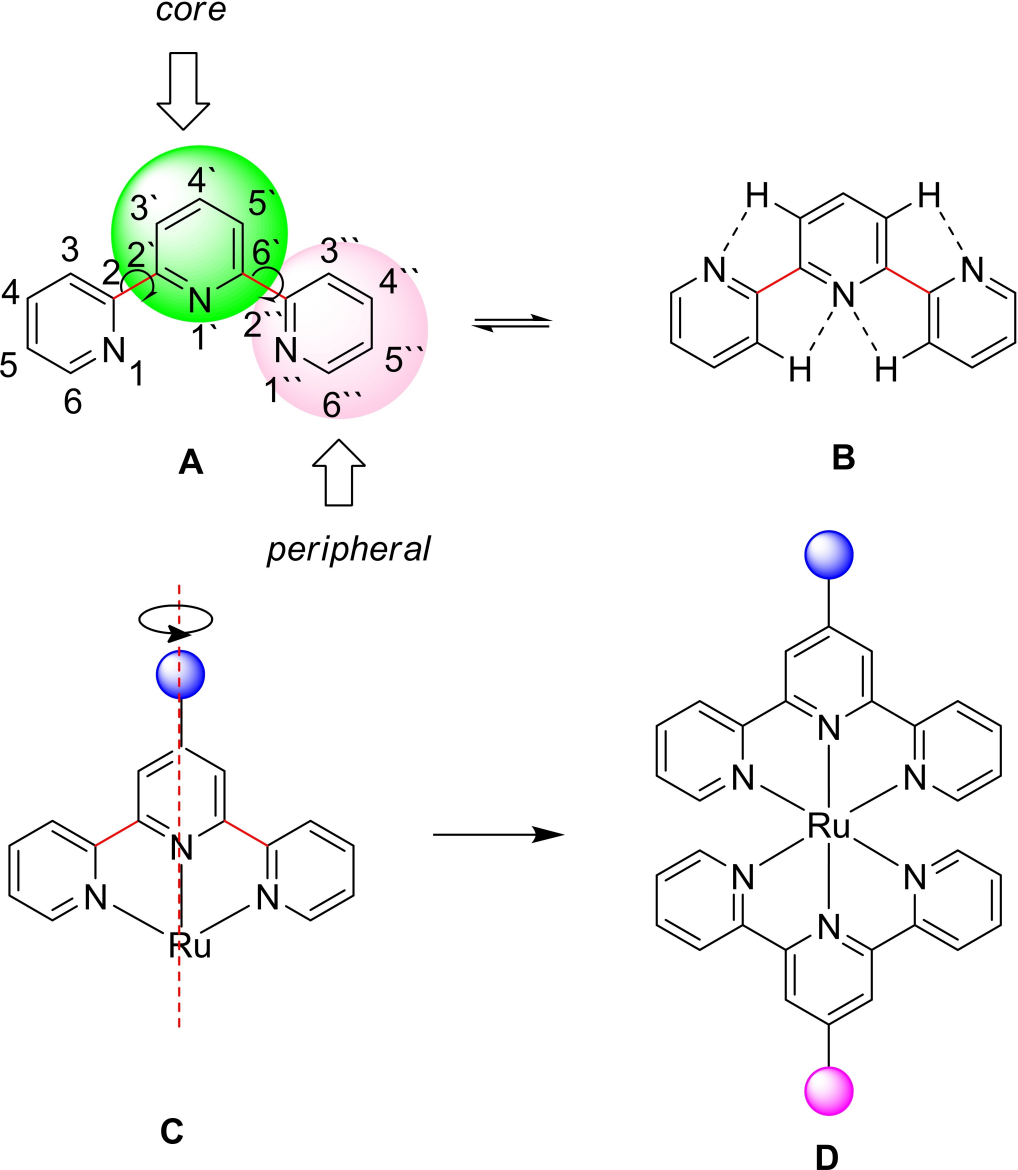
2,2‘ : 6‘,2“‐Terpyridines (tpy) (**A**); stabilization of the planar structure of tpy by the core pyridine ring through intramolecular hydrogen bonds (**B**); the stability and symmetry of the synthesized Ru(II) complex (**C**); and the designed structure of bis(terpyridine) Ru(II) complexes with different functionalization at the 4‘‐position (**D**).

The design and synthesis of new metal‐organic coordination compounds as metallotherapeutic drugs have garnered significant interest due to their multiple therapeutic functionalities, which are often not achievable with traditional organic molecules.[^1^, ^2^] Polypyridyl ruthenium complexes, in particular, are pivotal in therapeutic developments due to their unique characteristics, such as rapid ligand exchange, different oxidation states, and ruthenium's capability to imitate iron in binding to specific biological targets. Consequently, this class of metal complexes exhibits high biological activity, including DNA binding, anti‐inflammatory, antimicrobial, and anticancer properties.[^3^, ^4^, ^5^, ^6^, ^7^, ^8^] In particular, 2,2‘ : 6‘,2“‐terpyridines (terpy or tpy) (Figure 1A) as N‐heterocycles are of great significance because of their exceptional ability to form stable complexes. The presence of three connected pyridine units, which contain three tertiary ring nitrogen donor atoms, grants these ligands a high chelating ability.^[9]^ The core pyridine ring stabilizes the planar structure through intramolecular hydrogen bonds (CH…N) involving core and peripheral pyridine rings, as shown in structure B (Figure 1B).^[10]^ These nitrogen donor atoms form M–N_pyridine_ bonds in transition metal complexes, where the σ‐donor/π‐acceptor nature of these bonds provides high stability via dπ–pπ* back bonding.^[11]^ Moreover, 2,2‘ : 6‘,2“‐terpyridine ligands form octahedral geometric structures upon coordination with metal centers using the ‹tpy–M^II^–tpy› connection, leading to M^II^‐bis‐terpyridine complexes. This family of rigid, well‐defined complexes is typically synthesized through two consecutive steps. The incorporation of two tpy units to the metal ion center results in fascinating photophysical characteristics and enhances the therapeutic activity of these complexes.[^12^, ^13^]

Currently, many available non‐steroidal anti‐inflammatory drugs (NSAIDs) demonstrate high therapeutic efficacy by selectively inhibiting COX‐2 (coxibs) without affecting COX‐1 enzymatic activity.[^14^, ^15^, ^16^] However, despite their selective inhibition, potential cardiovascular side effects have brought these drugs under scrutiny.[^17^, ^18^] Additionally, inflammation can promote bacterial growth due to fluid accumulation in injured areas, necessitating the simultaneous prescription of separate anti‐inflammatory and antimicrobial drugs, complicating NSAID therapy.^[19]^ Chronic inflammation also predisposes individuals to cancer development and tumor progression, and treatment resistance is particularly facilitated by chronic inflammation.[^20^, ^21^] Furthermore, inflammation is a key feature of COVID‐19.^[22]^ Given these challenges, designing new, more potent, and safer compounds with combined anti‐inflammatory, antimicrobial, and anticancer effects is of paramount importance. Ruthenium complexes have established themselves as active metallochemotherapeutic agents, not only as derivatives of NSAIDs but also in exhibiting antimicrobial activity against drug‐resistant pathogens.[^3^, ^23^, ^24^]

The development and antibacterial activity of 2,2‘ : 6‘,2“‐terpyridine and bis(terpyridine) metal complexes [metal=ruthenium (II) and rhodium (III)] have been achieved, with some complexes showing superior activity compared to commercially available antibiotics such as kanamycin and streptomycin (Figure 1C).^[25]^ Functionalization of bis‐terpyridine at the 4‘‐position enables the design of systems with donor and acceptor groups in opposite directions (Figure 1D). Most NSAIDs contain carboxylic groups as hydrophilic elements and aromatic rings as lipophilic groups, creating an amphiphilic structure. This amphiphilic nature is crucial for the synergistic effect on cellular uptake and phototoxicity, and for the permeation through bacterial cell walls.^[26]^

In light of these aspects, and continuing our previous work on synthesizing 2,2‘ : 6‘,2“‐terpyridine Ru(II) complexes as anti‐inflammatory agents,^[27]^ this study aims to synthesize new 4‘‐functionalized bis(terpyridine) Ru(II) complexes with improved biological activity. In this regard, new heteroleptic Ru(II)‐complexes have been successfully synthesized in two steps, featuring one ligand as 4‐(2,2‘ : 6‘,2“‐terpyridine‐4‘‐yl) benzoic acid), and the second ligand as either 4‘‐(2‐thienyl)‐2,2‘ : 6‘,2“‐terpyridine, 4‘‐(3,4‐dimethoxyphenyl)‐2,2‘ : 6‘,2“‐terpyridine, or 4‘‐(4‐dimethylaminophenyl)‐2,2‘ : 6‘,2“‐terpyridine. The anti‐inflammatory, cytotoxic, and antimicrobial activities of these synthesized Ru(II) complexes were systematically evaluated, revealing how variations in the 4′‐substituents of the terpyridine motif affect their biological activity. Notably, these complexes demonstrate a wide range of robust biological activities. Furthermore, they have low haemolytic potential, effectively inhibit heat‐induced albumin denaturation to reduce inflammation, selectively target cancer cells, and possess broad‐spectrum antimicrobial properties. More importantly, the Ru(II) complex featuring a 3,4‐dimethoxyphenyl group on the terpyridine stands out as the most promising, exhibiting the highest anti‐inflammatory activity and remarkable antimicrobial effectiveness against various pathogens.

## Experimental Section

### Chemicals and Solvents

All chemicals including 2‐acetylpyridine (Sigma‐Aldrich, ≥99 %), thiophene‐2‐carbaldehyde (Sigma‐Aldrich, 98 %), 3,4‐dimethoxybenzaldehyde (Alfa‐Aesar, 99 %), 4‐(dimethylamino)benzaldehyde (Sigma‐Aldrich, 98 %) or 4‐methylbenzaldehyde (Across, 97 %), ruthenium(III) chloride hydrate (Sigma‐Aldrich, ≥99.9 %), ammonium hexafluorophosphate (NH_4_PF_6_) (Thermo Scientific Chemicals, 99.5 %) were utilized as received. All solvents were of HPLC grade and used directly without further purification.

### Characterization

^1^H NMR and ^13^C NMR spectra were obtained using Bruker 500 MHz instruments using the residual signals for CDCl_3_ at 7.26 and 77.0 ppm and for DMSO‐*d*_6_ at 2.50 and 39.4 ppm as internal references for ^1^H and ^13^C, respectively. High‐resolution mass spectrometry (HRMS) was conducted on an asolariX (Bruker Daltonik) with a 7.0 T superconducting magnet and interfaced to an Apollo II Dual ESI/MALDI source. UV–vis spectra were captured with a PerkinElmer Lambda 750 UV‐vis spectrophotometer, while emission spectra were measured using a FP‐6500 spectrofluorometer (Jasco, Japan).

### Synthesis

#### Synthesis of the Ligands

The ligands 4′‐(*p*‐tolyl)‐2,2′ : 6′,2′′‐terpyridine, 4‘‐(4‐dimethylaminophenyl)‐2,2‘ : 6‘,2“‐terpyridine, 4′‐(2‐thienyl)‐2,2′ : 6′,2′′‐terpyridine, and 4‘‐(3,4‐dimethoxyphenyl)‐2,2‘ : 6‘,2“‐terpyridine were synthesized following established literature methods.[^28^, ^29^] Detailed synthesis procedures are demonstrated in the supporting information. ^1^H NMR and ^13^C NMR spectra of the ligands are displayed in Figure S1.

#### Synthesis of the Ru(II) Complexes

The Ru(tpy)Cl_3_ complexes were synthesized as following; terpyridine ligand (4′–(4‐dimethylaminophenyl)‐2,2‘ : 6‘,2“‐terpyridine, 4′‐(2‐thienyl)‐2,2′ : 6′,2′′‐terpyridine, or 4‘‐(3,4‐dimethoxyphenyl)‐2,2‘ : 6‘,2“‐terpyridine) was reacted with RuCl_3._3H_2_O in dry methanol for 6 h. After the reaction, the methanol was removed by evaporation under reduced pressure. The product was then filtered with cold methanol, and the resulting solid was dried. This solid was subsequently reacted with the ligand 4‘‐([2,2′ : 6′,2′′‐terpyridine]‐4′‐yl)benzoic acid in 10 ml of DMF for 6 h. The product was precipitated by adding the reduced volume of the DMF reaction mixture to an excess of saturated aqueous NH₄PF₆ solution. The resulting solid was filtered, washed with distilled water and diethyl ether, and dried under vacuum. The yields of (4′‐(thiophen‐2‐yl)‐2,2′ : 6′,2′′‐terpyridine) (4‐([2,2′ : 6′,2′′‐terpyridin]‐4′‐yl)benzoic acid) Ru(II)di(hexafluoro‐λ^6^‐phosphane), (4′‐(3,4‐dimethoxyphenyl)‐2,2′ : 6′,2′′‐terpyridine) (4‐([2,2′ : 6′,2′′‐terpyridin]‐4′‐yl)benzoic acid) Ru(II)di(hexafluoro‐λ^6^‐phosphane), and (4‐([2,2′ : 6′,2′′‐terpyridin]‐4′‐yl)‐N,N‐dimethylaniline) (4‐([2,2′ : 6′,2′′‐terpyridin]‐4′‐yl)benzoic acid) Ru(II)di(hexafluoro‐λ^6^‐phosphane) are 62 %, 65 %, and 70 %, respectively. The NMR spectra of the Ru(II) complexes are displayed in Figure S2, and the mass spectra are demonstrated in Figure S3.

#### (4′‐(Thiophen‐2‐yl)‐2,2′ : 6′,2′′‐terpyridine) (4‐([2,2′ : 6′,2′′‐terpyridin]‐4′ yl)benzoic acid) Ru(II) di(hexafluoro‐λ^6^‐phosphane)

^1^H NMR (400 MHz, DMSO) δ 9.52 (s, 2H, C_3‘_‐*H*), 9.34 (s, 2H, C_5‘_‐*H*), 9.14 (dd, *J*=21.7, 8.1 Hz, 4H, C_4_‐*H*, C_4“_‐*H*), 8.44 (dd, *J*=18.8, 5.4 Hz, 4H, C_5_‐*H*, C_5“_‐*H*), 8.20 (d, *J*=7.9 Hz, 4H, C_6_‐*H*, C_6“_‐*H*)), 8.07 (t, *J*=7.8 Hz, 4H, C_3_‐*H*, C_3“_‐*H*), 8.03 (d, *J*=5.1 Hz, 2H, Ar‐*H*), 7.64 – 7.53 (m, 2H, thiophene‐*H*), 7.53 – 7.42 (m, 2H, Ar‐*H*), 7.29 (s, 1H, thiophene‐*H*) ppm.

^13^C NMR (100 MHz, DMSO) δ 168.10, 158.54, 158.28, 155.55, 155.49, 152.71, 147.36, 141.39, 140.48, 138.52, 130.74, 130.39, 129.73, 129.24, 128.30, 128.17, 127.28, 125.38, 121.58, 119.89 ppm.

MS (MALDI) m/z calcd for [C_41_H_28_F_12_N_6_O_2_P_2_RuS] (M)^+^: 1060.0322, found 1060.2122.

#### (4‐([2,2′ : 6′,2′′‐Terpyridin]‐4′‐yl)‐N,N‐dimethylaniline) (4‐([2,2′ : 6′,2′′terpyridin]‐4′‐yl)benzoic acid) Ru(II) di(hexafluoro‐λ^6^‐phosphane)

^1^H NMR (400 MHz, DMSO) δ 9.44 (s, 2H, C_3‘_‐*H*), 9.30 (s, 2H, C_5‘_‐*H*), 9.04 (dd, *J*=15.2, 8.10 Hz, 4H, C_4_‐*H*, C_4“_‐*H*), 8.40 (d, *J*=8.20 Hz, 4H, C_5_‐*H*, C_5“_‐*H*), 8.29 (d, *J*=8.8 Hz, 4H, C_6_‐*H*, C_6“_‐*H*)), 8.17 (d, *J*=8.0 Hz, 4H, C_3_‐*H*, C_3“_‐*H*), 7.98 (d, *J*=5.1 Hz, 2H, Ar‐*H*), 7.47 (m, 2H, Ar‐*H*), 7.30–7.10 (m, 2H, Ar‐*H*), 6.93 (d, *J*=8.9 Hz, 2H, Ar‐*H*), 3.05 (s, 6H, N(C*H*_3_)_2_) ppm.

^13^C NMR (101 MHz, DMSO) δ 167.88, 162.79, 158.75, 158.56, 155.82, 155.03, 152.70, 152.19, 147.97, 138.42, 130.50, 129.08, 128.27, 128.01, 127.77, 125.30, 122.74, 121.67, 119.57, 112.59, 36.25, 31.24 ppm.

MS (MALDI) m/z calcd for [C_45_H_35_ F_12_N_7_O_2_P_2_Ru] (M)^+^: 1097.1179, found 808.1645 [M+1–2PF_6_^−^]. PF_6_^−^ units can be lost upon MALDI which is accompanied by electron capture and lower the mass by (145 for each PF_6_ unit).

#### (4′‐(3,4‐Dimethoxyphenyl)‐2,2′ : 6′,2′′‐terpyridine) (4‐([2,2′ : 6′,2′′‐terpyridin]‐4′ yl)benzoic acid) Ru(II) di(hexafluoro‐λ^6^‐phosphane)

^1^H NMR (400 MHz, DMSO) δ 9.53 (s, 2H, C_3‘_‐*H*), 9.41 (s, 2H, C_5‘_‐*H*), 9.16 (d, *J*=14.8 Hz, 4H, C_4_‐*H*, C_4“_‐*H*), 8.73 (d, *J*=19.9 Hz, 4H, C_5_‐*H*, C_5“_‐*H*), 8.42 (s, 4H, C_6_‐*H*, C_6“_‐*H*)), 8.08 (m, 4H, C_3_‐*H*, C_3“_‐*H*), 7.57 (s, 4H, Ar‐*H*), 7.31 (s, 3H, Ar‐*H*), 4.08 (s, 3H, −OC*H*_3_), 3.97 (s, 3H, −OC*H*_3_) ppm.

^13^C NMR (101 MHz, DMSO) δ 171.65, 158.53, 156.19, 155.58, 155.35, 155.28, 152.53, 151.40, 149.87, 147.64, 147.16, 138.51, 138.44, 138.01, 137.35, 130.46, 129.04, 128.19, 127.40, 126.78, 125.27, 125.08, 121.62, 121.46, 121.09, 118.45, 112.63, 111.63, 56.61, 56.25 ppm.

MS (MALDI) m/z calcd for [C_45_H_34_ F_12_N_6_O_4_P_2_Ru] (M)^+^: 1114. 0969, found 1114.1991.

### Biological Activity Investigations

Details of the hemolysis assay for assessing cytotoxicity, the in vitro anti‐inflammatory albumin denaturation assay, the crystal violet assay for evaluating cell viability, and the antimicrobial activity tests are provided in the supporting information.

## Results and Discussion

### Synthesis

**Scheme 1:**
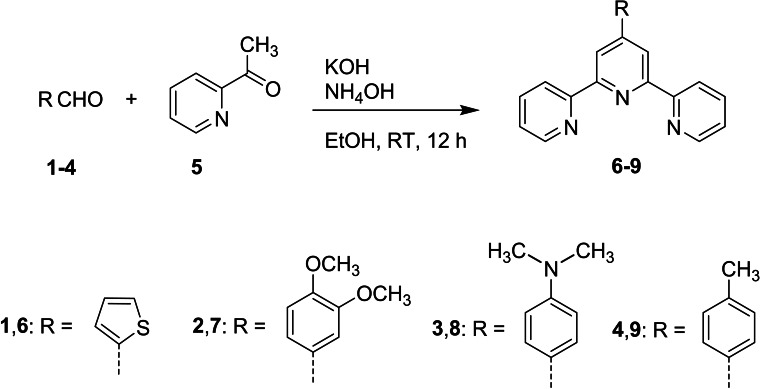
Synthetic route for the synthesis of the terpyridyl ligands

**Scheme 2:**
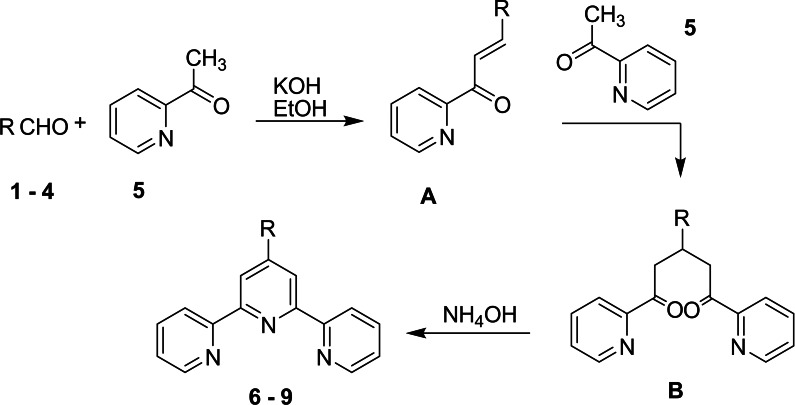
Detailed synthetic steps for the formation of terpyridine, highlighting the key intermediates

Although several synthetic routes have been proposed for the synthesis of 2,2‘ : 6‘,2“‐terpyridine derivatives (tpy),[^30^, ^31^, ^32^, ^33^] the most common approach involves first synthesizing the intermediate 1,5‐bis(pyridine‐2‐yl)pentane‐1,5‐dione, which is then cyclized to form the central pyridine ring using an ammonia source.[^34^, ^35^, ^36^] Scheme 1 exhibits the standard approach employed for the synthesis of four terpyridine derivatives; 4‘‐(2‐thienyl)‐2,2‘ : 6‘,2“‐terpyridine **6**, 4‘‐(3,4‐dimethoxyphenyl)‐2,2‘ : 6‘,2“‐terpyridine **7**, 4‘‐(4‐dimethylaminophenyl)‐2,2‘ : 6‘,2“‐terpyridine **8** and 4‘‐(4‐tolyl)‐2,2‘ : 6‘,2“‐terpyridine **9**. This method is regarded as a one‐pot reaction, involving the condensation of 2‐acetylpyridine **5** with thiophene‐2‐carbaldehyde **1**, 3,4‐dimethoxybenzaldehyde **2**, 4‐(dimethylamino)benzaldehyde **3** or 4‐methylbenzaldehyde **4** under basic conditions to obtain terpyridine derivatives **6**–**9** before adding NH_4_OH (Scheme 1). The postulated mechanism of formation of **6**–**9** is clearly illustrated in Scheme 2. The synthesis begins with the Kröhnke condensation of 2‐acetylpyridine **5** with aldehyde derivatives **1**–**4** to produce the enone (chalcone) **A**. In the second step, a Michael addition of a second molecule of 2‐acetylpyridine **5** to the condensation product **A** forms the 1,5‐dione **B**. Finally, cyclization is achieved by treating **B** with ammonium hydroxide, which provides the ammonia needed for ring closure, resulting in the formation of the central pyridine unit and yielding the desired terpyridine ligands **6–9** (Scheme 2).

**Scheme 3:**
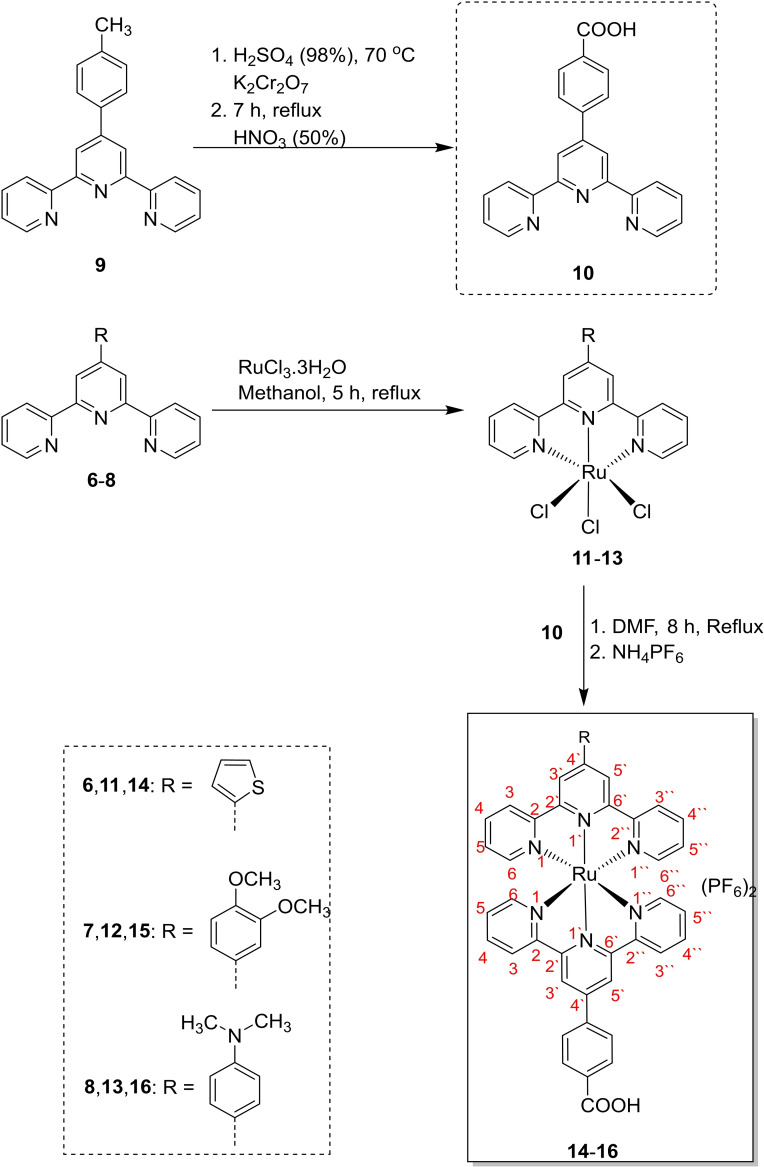
Synthetic steps of the heteroleptic ruthenium (II) complexes

The heteroleptic complexes [Ru(4‘‐(2‐thienyl)‐2,2‘ : 6‘,2“‐terpyridine)(4‐(2,2‘ : 6‘,2“‐terpyridine‐4‘‐yl) benzoic acid)]^2+^ **14**, [Ru(4‘‐(3,4‐dimethoxyphenyl)‐2,2‘ : 6‘,2“‐terpyridine)(4‐(2,2‘ : 6‘,2“‐terpyridine‐4‘‐yl) benzoic acid)]^2+^ **15** and [Ru(4‘‐(4‐dimethylaminophenyl)‐2,2‘ : 6‘,2“‐terpyridine)(4‐(2,2‘ : 6‘,2“ terpyridine‐4‘‐yl) benzoic acid]^2+^ **16** were successfully prepared in two steps, as illustrated in Scheme 3. Initially, terpyridine ligands **6**–**8** are reacted with RuCl_3_.3H_2_O in DMF as a solvent for 5 h to generate [Ru(4‘‐(2‐thienyl)‐2,2‘ : 6‘,2“‐terpyridine)Cl_3_] **11**, [Ru(4‘‐(3,4‐dimethoxyphenyl)‐2,2‘ : 6‘,2“‐terpyridine)Cl_3_] **12** and [Ru(4‘‐(4‐dimethylaminophenyl)‐2,2‘ : 6‘,2“‐terpyridine)Cl_3_] **13**. In the subsequent step, the complexes **11**–**13** are refluxing with 4‐(2,2‘ : 6‘,2“‐terpyridine‐4‘‐yl) benzoic acid **10** (formed *via* oxidation of 4‘‐(4‐Methylphenyl)‐2,2‘ : 6‘,2“‐terpyridine **9** using potassium dichromate and sulfuric acid) in DMF as a solvent for 8 h followed by addition of ammonium hexafluorophosphate to obtain the desired heteroleptic complexes **14**–**16** (Scheme 3).[^37^, ^38^] The Ru(II) complexes Ru(4‘‐(2‐thienyl)‐2,2‘ : 6‘,2“‐terpyridine)(4‐(2,2‘ : 6‘,2“‐terpyridine‐4‘‐yl) benzoic acid)](PF_6_)_2_, [Ru(4‘‐(3,4‐dimethoxyphenyl)‐2,2‘ : 6‘,2“‐terpyridine)(4‐(2,2‘ : 6‘,2“‐terpyridine‐4‘‐yl) benzoic acid)](PF_6_)_2_ and [Ru(4‘‐(4‐dimethylaminophenyl)‐2,2‘ : 6‘,2“‐terpyridine) (4‐(2,2‘ : 6‘,2“‐terpyridine‐4‘‐yl) benzoic acid](PF_6_)_2_ are termed as RuTBCTS, RuTBCTMeO, and RuTBCTN, respectively.

### Photophysical Characterizations

**Figure 2:**
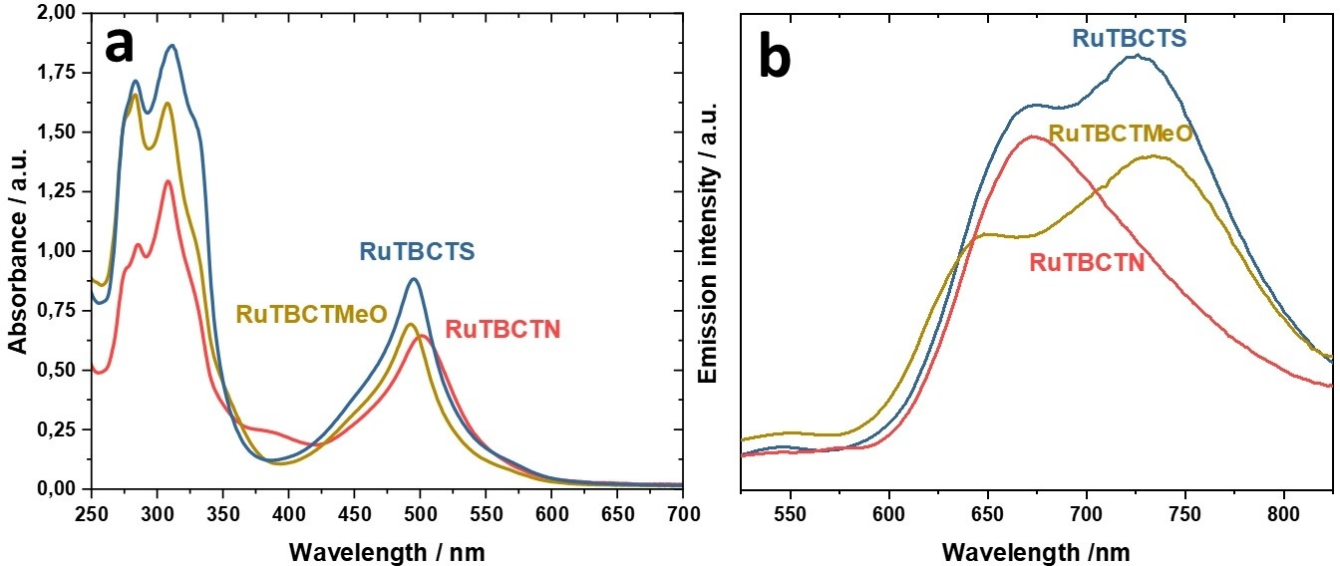
(a) Absorption and (b) emission spectra of RuTBCTN, RuTBCTMeO, and RuTBCTS recorded in acetonitrile.

The absorption spectra of the newly synthesized complexes RuTBCTN, RuTBCTMeO, and RuTBCTS in acetonitrile are illustrated in Figure 2a. In the visible regime, these spectra cover a wide range (400–600 nm) with major absorption peaks at 502 nm, 494 nm, and 495 nm, respectively, which contribute to their deep red color. These peaks are primarily correlated to spin‐allowed metal‐to‐ligand charge transfer (MLCT) transitions. The presence of this band in 4′‐substituted‐2,2′ ; 6′,2′′‐tpy Ru(II) complexes is characteristic of an octahedral geometry, matching the ^1^T_1(g)_ ← ^1^A_1(g)_ transition.[^39^, ^40^] The molar extinction coefficients of complexes RuTBCTN, RuTBCTMeO, and RuTBCTS are 3.14×10^4^, 3.42×10^4^, and 4.02×10^4^ M^−1^ cm^−1^, respectively. These high absorbance values are attributable to the coordination of two terpyridine (tpy) ligands per Ru(II) center, which enhances the overall π‐conjugation. The extended π‐conjugation is further facilitated by the 4‐dimethylaminophenyl, thiophenyl, or 4‐methoxyphenyl groups attached to the tpy ligands, as well as by the benzoic acid moiety on the other terpyridyl ligand. The absorption bands observed at wavelengths shorter than 400 nm are allocated to intraligand (π–π*) charge transitions of the terpyridine ligands. The molar extinction coefficients of the complexes RuTBCTN, RuTBCTMeO, and RuTBCTS at 310 nm are 6.29×10^4^, 7.97×10^4^, and 8.32×10^4^ M^−1^ cm^−1^, respectively.

The stability of these synthesized complexes was also tested via UV‐vis spectroscopy. The results revealed no significant spectral changes after one week at room temperature (25 °C), confirming their stability in their coordinating mode during biological activity investigations.

The emission spectra of the Ru(II) complexes in acetonitrile at 25 °C are demonstrated in Figure 2b. The proposed Ru(II) complexes display emission spectra that span a significant portion of the visible light regime upon excitation of the MLCT bands. The MLCT bands for the complexes are observed at approximately 650 nm, 672 nm, and 670 nm for RuTBCTN, RuTBCTS, and RuTBCTMeO, respectively indicating the presence of a “Ru (terpy)” moiety, influenced by the chromophoric behavior of conjugated substituents at the 4′‐position. Complexes RuTBCTMeO and RuTBCTS show two bands in their emission spectra. For RuTBCTMeO, there is a lower intensity band at approximately 650 nm and a slightly higher intensity band at around 735 nm. Similarly, RuTBCTS displays a lower intensity band at around 670 nm and another band with slightly higher intensity at approximately 730 nm. The luminescence properties of both complexes can be explained by the simultaneous phosphorescence from two non‐degenerate _3_MLCT excited states, each localized on different ligands within the heteroleptic complex.[^41^, ^42^]

### Biological Investigations

#### Haemolysis Assay

**Figure 3:**
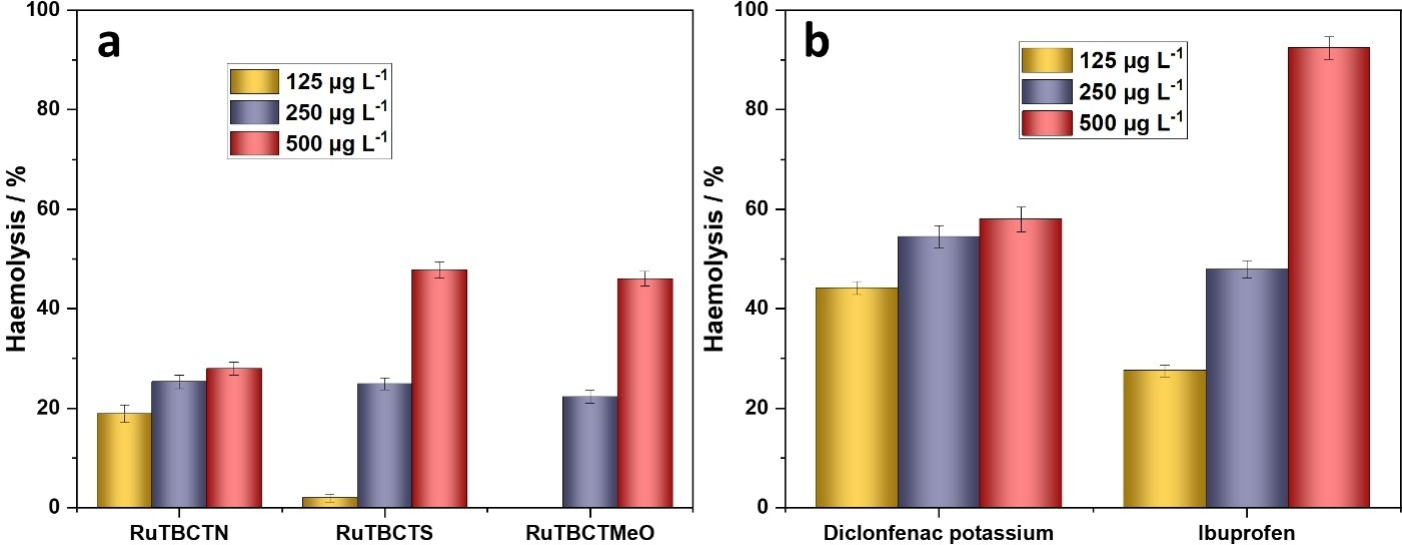
Effect of the (a) Ru(II) complexes and (b) standard drugs on the haemolysis of red blood cells (RBCs)

Toxicity assessment is a crucial step in drug development, ensuring the safety and therapeutic potential of new compounds.[^43^, ^44^] We evaluated the toxicity of the proposed Ru(II) complexes through an in vitro hemolysis assay, benchmarked against diclofenac potassium and ibuprofen, as standard drugs (Figures 3a and b). Hemolysis, the rupture of erythrocytes and subsequent release of haemoglobin, serves as an indicator of cytotoxicity.^[45]^ Hemolytic activity is categorized by the mortality rate: non‐toxic (0–9 %), slightly toxic (10–49 %), toxic (50–89 %), and highly toxic (90–100 %).[^46^, ^47^]

The Ru(II) complexes – RuTBCTN, RuTBCTS, and RuTBCTMeO – show slightly different toxicity profiles (Figure 3a). At 125 mg L^−1^, only RuTBCTN is slightly toxic, while RuTBCTS and RuTBCTMeO remain non‐toxic. At 250 and 500 mg L^−1^, all three complexes are slightly toxic. In comparison, the standard drugs show notable levels of haemolysis, ranging from 54.5 % to 58.0 % for diclofenac potassium and 47.9 % to 92.4 % for ibuprofen at concentrations between 250 and 500 mg L^−1^ (Figure 3b), consistent with their established toxicity profiles.^[55]^ In contrast, the proposed compounds in this study exhibit much lower haemolytic activity, indicating greater safety margins compared to these drugs. These results emphasize the promising potential of these Ru(II) complexes for further pharmacological studies.

#### Inhibition of Albumin Denaturation and Anti‐inflammatory Properties

**Figure 4:**
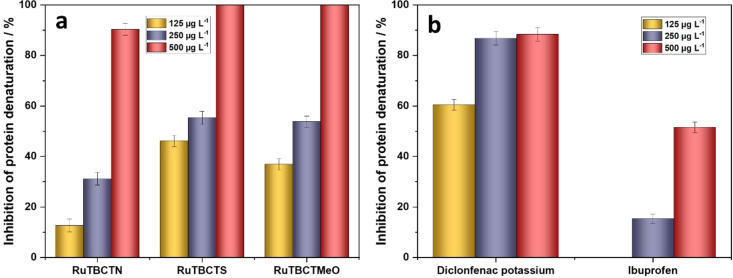
Activity of (a) the proposed Ru(II) complexes and (b) standard drugs in inhibiting heat‐induced albumin denaturation

To evaluate the in vitro anti‐inflammatory activity of the Ru(II) complexes, their ability to inhibit heat‐induced albumin denaturation was investigated and compared to standard drugs (ibuprofen and diclofenac potassium). The results are demonstrated in Figures 4a and b. At a concentration of 125 μg mL^−1^, the Ru(II) complexes RuTBCTN, RuTBCTS, and RuTBCTMeO show inhibitory activities of 12.6 %, 46.25 %, and 36.9 %, respectively. The inhibitory activity of the complexes increases significantly with increasing their amount, reaching a maximum of 90.3 % for RuTBCTN and 100 % for RuTBCTS and RuTBCTMeO at 500 μg mL^−1^ (Figure 4a). The diclofenac potassium and ibuprofen inhibit heat‐induced albumin denaturation by 88.37 % and 51.5 %, respectively, at 500 μg mL^−1^, which is much lower than the proposed complexes (Figure 4b). Furthermore, the complexes with thiophene and 3,4‐dimethoxyphenyl substituted groups are more effective in the inhibition of albumin denaturation than that of 4‐dimethylaminophenyl.

#### Anticancer Activity

**Figure 5:**
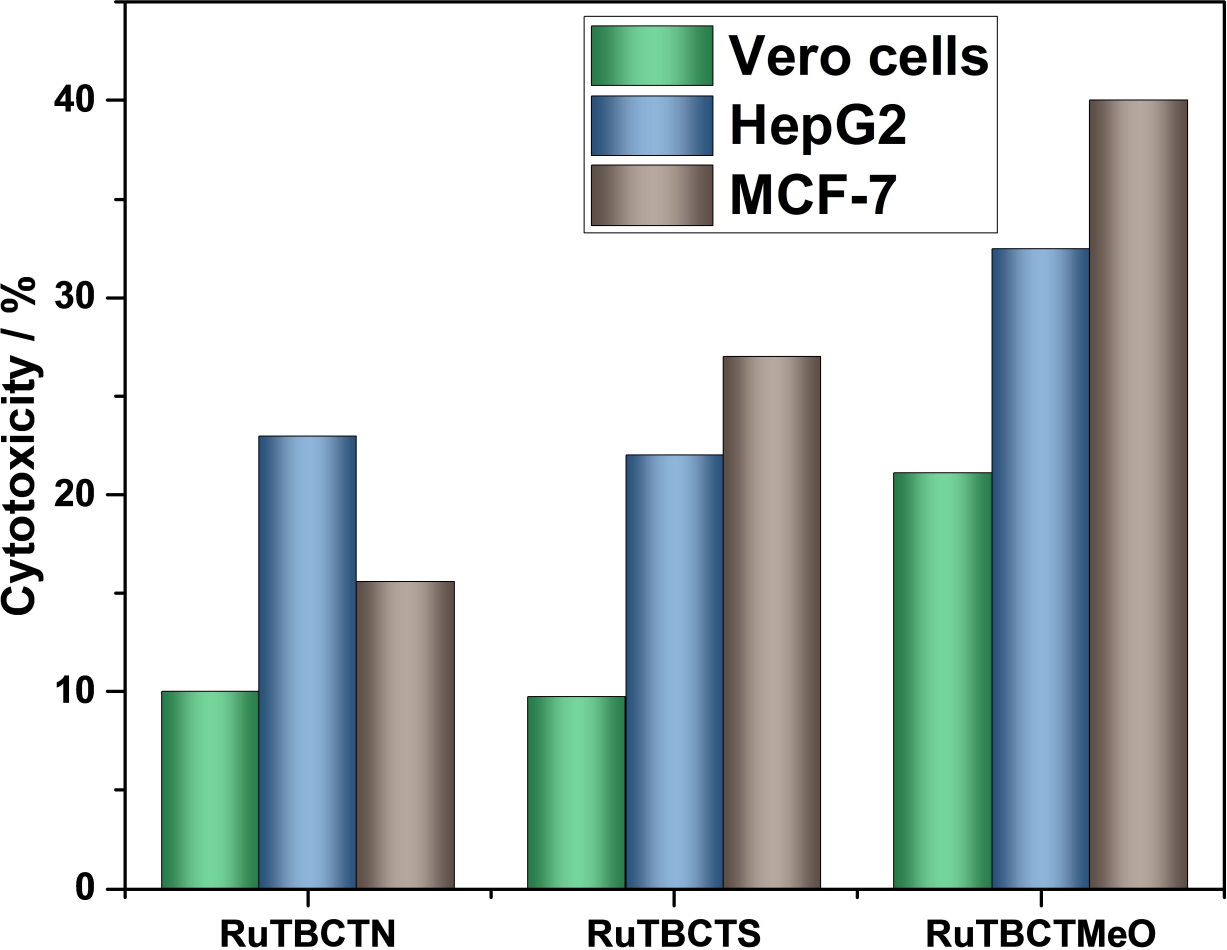
In vitro cytotoxic activity of the proposed Ru(II) complexes against Vero cells, HepG2, and MCF‐7 cell lines

The in vitro cytotoxicity of the synthesized Ru(II) complexes was investigated using the crystal violet dye reduction assay. This assay was carried out using two human cancer cell lines (HepG2 and MCF‐7) and one normal cell line derived from monkey kidney cells (Vero cells). DMSO was used as a solvent at concentrations below 2.5 % (v/v), which are considered non‐toxic to the cells. The concentration of each Ru(II) complex was 200 mg L^−1^. The results are presented in Figure 5. Cytotoxic activity is categorized as follows: remarkable cytotoxic activity (>75 % of the cell population), moderate cytotoxic activity (40–75 % of the cell population), low cytotoxic activity (0.1–40 % of the cell population), and no cytotoxic activity (0 % of the cell population).^[48]^ The Ru(II) complexes RuTBCTN, RuTBCTS, and RuTBCTMeO display varying levels of cytotoxic activity, influenced by the moieties on the 4′‐substituted‐2,2′ : 6′,2′′‐tpy ligands. The complex with the 3,4‐dimethoxyphenyl moiety exhibits the highest cytotoxicity, followed by the complex with the thiophenyl moiety, and lastly, the one with the 4‐dimethylaminophenyl moiety. These complexes also show higher selectivity against cancer cells compared to normal cells.

RuTBCTMeO demonstrates moderate cytotoxic activity against MCF‐7 cells (41.0 %) and lower cytotoxic activity towards HePG2 cells (32.5 %) and Vetro cells (21.1 %). In comparison, the RuTBCTS and RuTBCTN complexes show low cytotoxic activity across all the investigated cell lines. Typically, Ru(II) terpyridine complexes exhibit cytotoxicity through several mechanisms: (i) robust binding to nucleic acids and proteins, (ii) ligand exchange processes, (iii) redox cycling between oxidation states II and III, and (iv) the ability of Ru(II) complexes to mimic iron in their interaction with biological molecules.[^1^, ^5^, ^27^, ^49^, ^50^, ^51^, ^52^] Furthermore, terpyridine derivatives can induce DNA damage, disrupt calcium signaling, block receptor channels, and interact with proteins.[^53^, ^54^]

#### Antimicrobial Activity

Table 1 displays the antimicrobial activity of the proposed Ru(II) complexes in comparison with the standard drugs tetracycline and cycloheximide. The complexes were tested against Aspergillus niger NRRL A 326 (fungus), Candida albicans ATCC 10231 (yeast), Pseudomonas aeruginosa (Gram‐negative bacterium), and Staphylococcus aureus ATCC 6538 (Gram‐positive bacterium). Notably, the RuTBCTMeO complex exhibits the highest overall antimicrobial activity among the Ru(II) complexes, demonstrating significant broad‐spectrum efficacy against all tested microorganisms. In contrast, the RuTBCTS complex shows high selectivity for Aspergillus niger, with no activity against Candida albicans, Pseudomonas aeruginosa, and Staphylococcus aureus. The RuTBCTN complex exhibits comparable antimicrobial activity to RuTBCTMeO against Candida albicans, Pseudomonas aeruginosa, and Staphylococcus aureus but lacks activity against Aspergillus niger. Overall, the RuTBCTMeO complex emerges as a promising candidate for antimicrobial applications, showing superior activity compared to the standard drugs tetracycline and cycloheximide, as well as some previously reported Ru(II) terpyridine complexes.^[55]^

**Table 1:** Antimicrobial performance of the proposed Ru(II) complexes compared tetracycline and cycloheximide.

Compound	Clear zone/mm			
	***Aspergillus** niger*	***Candida** albicans*	***Pseudomonas** aeruginosa*	***Staphylococcus** aureus*
Tetracycline	0	28	34	36
Cycloheximide	39	0	0	0
RuTBCTN	0	21	23	18
RuTBCTS	25	0	0	0
RuTBCTMeO	32	23	24	21

## Conclusions

This study demonstrates the synthesis and characterization of three novel 4′‐functionalized bis(terpyridine) Ru(II) complexes, (4′‐(thiophen‐2‐yl)‐2,2′ : 6′,2′′‐terpyridine) (4‐([2,2′ : 6′,2′′‐terpyridin]‐4′yl)benzoic acid) Ru(II) di(hexafluoro‐λ^6^‐phosphane) (RuTBCTS), (4‐([2,2′ : 6′,2′′‐terpyridin]‐4′‐yl)‐N,N‐dimethylaniline) (4‐([2,2′ : 6′,2′′terpyridin]‐4′‐yl)benzoic acid) Ru(II) di(hexafluoro‐λ^6^‐phosphane) (RuTBCTN), and (4′‐(3,4‐dimethoxyphenyl)‐2,2′ : 6′,2′′‐terpyridine) (4‐([2,2′ : 6′,2′′‐terpyridin]‐4′yl)benzoic acid) Ru(II) di(hexafluoro‐λ^6^‐phosphane) (RuTBCTMeO). The absorption spectra of the complexes RuTBCTN, RuTBCTMeO, and RuTBCTS in acetonitrile show major peaks at 502 nm, 494 nm, and 495 nm, respectively, attributed to MLCT transitions, indicating octahedral geometry. The high molar extinction coefficients of the Ru(II) complexes result from the extensive π‐conjugation of the tpy ligands. Emission spectra exhibit MLCT bands around 650 nm, 672 nm, and 670 nm for RuTBCTN, RuTBCTS, and RuTBCTMeO, respectively, with RuTBCTMeO and RuTBCTS showing a second emission band at 735 nm and 730 nm, respectively, likely due to phosphorescence from two non‐degenerate ^3^MLCT excited states. Interestingly, these complexes exhibit diverse and robust biological activities. Biological assessments reveal low haemolytic potential, effective anti‐inflammatory activity via the inhibition of thermal‐induced albumin denaturation, selective cytotoxicity against cancer cells, and broad‐spectrum antimicrobial effects. Particularly, the Ru(II) complex with 3,4‐dimethoxyphenyl moiety on the terpyridine emerges as a promising candidate with the highest anti‐inflammation activity and exceptional antimicrobial efficacy against multiple pathogens. These findings underscore the potential of 4′‐functionalized bis(terpyridine) Ru(II) complexes as versatile therapeutic agents, addressing critical challenges in inflammation, cancer therapy, and infectious diseases. Further research is needed to optimize their pharmacological properties and elucidate their mechanisms of action to facilitate clinical translation and application.

## Conflict of Interests

The authors declare no conflict of interest.

1

## Supporting information

As a service to our authors and readers, this journal provides supporting information supplied by the authors. Such materials are peer reviewed and may be re‐organized for online delivery, but are not copy‐edited or typeset. Technical support issues arising from supporting information (other than missing files) should be addressed to the authors.

Supporting Information

## Data Availability

Data will be made available on request.
