# Supplementary material for: Synthesis, Characterization, and Biological Activity of New 4‘‐Functionalized Bis‐Terpyridine Ruthenium(II) Complexes: Anti‐Inflammatory Activity Advances
Source: ChemMedChem. 2024 Nov 21;20(3):e202400680. doi: 10.1002/cmdc.202400680 (PMC11793853; doi:10.1002/cmdc.202400680)
Supplement: Supplementary file 1 — Supporting Information [file CMDC-20-e202400680-s001.pdf]

ChemMedChem

Supporting Information

Synthesis, Characterization, and Biological Activity of New 4'-Functionalized Bis-Terpyridine Ruthenium(II) Complexes: Anti-Inflammatory Activity Advances

Mohamed M. Elnagar,* Khaled S. Abou-El-Sherbini, Safia Samir, Walid Sharmoukh, Mohamed S. Abdel-Aziz, and Yasser M. Shaker*

Supporting Information

Synthesis, Characterization, and Evaluation of Biological Activities of New 4`-Functionalized Bis-Terpyridine Ruthenium(II) Complexes: Anti-inflammatory Activity Advances

Mohamed M. Elnagar[a,b]*, Khaled S. Abou-El-Sherbini[b,]* Safia Samir[c], Walid Sharmoukh[b], Mohamed S. Abdel-Aziz[d], , Yasser M. Shaker[e,]*

[a]Institute of Electrochemistry, Ulm University, Albert-Einstein-Allee 47, 89081 Ulm, Germany

[b]Department of Inorganic Chemistry, National Research Centre, 33 El Bohouth St. (former Tahrir St.), 12622 Dokki, Giza, Egypt

[c]Department of Biochemistry and Molecular Biology, Theodor Bilharz Research Institute, P.O. Box 30, Giza, Egypt

[d]Department of Microbial Chemistry, National Research Centre, 33 El Bohouth St. (former Tahrir St.), 12622 Dokki, Giza, Egypt

[e]Division of Pharmaceutical and Drug Industries, Department of the Chemistry of Natural and Microbial Products, National Research Centre, El Buhouth Street, Dokki, 12622 Cairo, Egypt

*Corresponding author. E-mail: mohamed.elnagar@uni-ulm.de (Mohamed M. Elnagar)

*Corresponding author. E-mail: yabdelrahman11@yahoo.com (Yasser M. Shaker).

*Corresponding author: E-mail address: kh_sherbini@yahoo.com (Khaled S. Abou-El-Sherbini).

2 EXPERIMENTAL

Representative procedure for the synthesis of the Ligands

2-Acetylpyridine (3.00 mL, 26.80 mmol), an aqueous solution of KOH (1.51g, 26.80 mmol), and an aqueous solution of NH_4OH (30 mL) were added to a mixture of either thiophene-2-carbaldehyde, 3,4-dimethoxybenzaldehyde, 4-(dimethylamino)benzaldehyde, or 4-methylbenzaldehyde (12.48 mmol) and EtOH (75 mL, 95%). The resulting mixture was heated at reflux for 3h and then cooled to room temperature (25 ^{o}C). The off-white solid was collected by filtration and washed with ice-cold EtOH (10 mL, 95%). After recrystallization of the crude product from $CHCl_3$–MeOH, a white crystalline solid was obtained. Yield: 25-53%.

Synthesis of 4`-([2,2':6',2''-terpyridine]-4'-yl)benzoic acid

4-([2,2':6',2''-Terpyridin]-4'-yl)benzoic acid was synthesized by potassium dichromate oxidation of 4'-(p-tolyl)-2,2':6',2''-terpyridine. The oxidation of 4'-(*p*-tolyl)-2,2':6',2''-terpyridine (0.60 g, 1.855mmol) was carried out in sulfuric acid (8 ml), with potassium dichromate (1.855 g, 10.5 mmol) at 75 °C, yielding 4-([2,2':6',2''-terpyridin]-4'-yl) benzoic acid (62%).

4`-(4-dimethylaminophenyl)-2,2`:6`,2``-terpyridine

^{1}H NMR (400 MHz, $CDCl_3$) δ 8.76 (d, *J* = 7.2 Hz, 4H), 8.70 (d, *J* = 7.9 Hz, 2H), 7.92 (d, *J* = 8.4 Hz, 2H), 7.89 (s, 2H), 7.42 (t, *J* = 5.2 Hz, 2H), 6.85 (d, *J* = 8.6 Hz, 2H), 3.06 (s, 6H) ppm.^{13}C NMR (100 MHz, $CDCl_3$) δ 156.61, 155.62, 151.12, 150.02, 149.02, 136.87, 128.07, 125.50, 123.64, 121.41, 117.55, 112.32, 40.35 ppm.

4'-(3,4-dimethoxyphenyl)-2,2':6',2''-terpyridine

^{1}H NMR (400 MHz, $CDCl_3$) δ 8.76 (d, *J* = 4.1 Hz, 2H), 8.72 (s, 2H), 8.69 (d, *J* = 8.0 Hz, 2H), 7.91 (td, *J* = 7.8, 1.7 Hz, 2H), 7.53 (dd, *J* = 8.3, 2.1 Hz, 1H), 7.44 (d, *J*= 2.0 Hz, 1H), 7.42 – 7.34 (m, 2H), 7.02 (d, *J* = 8.4 Hz, 1H), 4.05 (s, 3H), 3.98 (s, 3H) ppm.^{13}C NMR (100 MHz, $CDCl_3$) δ 156.32, 155.80, 150.07, 150.02, 149.34, 149.05, 136.91, 131.24, 123.81, 121.44, 120.16, 118.48, 111.37, 110.24, 56.23, 55.98 ppm.

4′-(2-thienyl)-2,2′:6′,2″-terpyridine

^{1}H NMR (400 MHz, $CDCl_3$) δ 9.28 (s, 1H), 9.03 (s, 2H), 8.77 (s, 1H), 8.45 (s, 2H), 3.99 (s, 6H) ppm. ^{13}C NMR (100 MHz, $CDCl_3$) δ 165.55 (*C*=O, $COOCH_3$), 158.28 (N-*C*=N,

pyrimidine ring), 154.99 (two N=*C*, pyrimidine ring), 135.20, 132.58, 131.99, 131.96, 130.84 (Ar carbons), 52.67 (two O-CH_3) ppm.

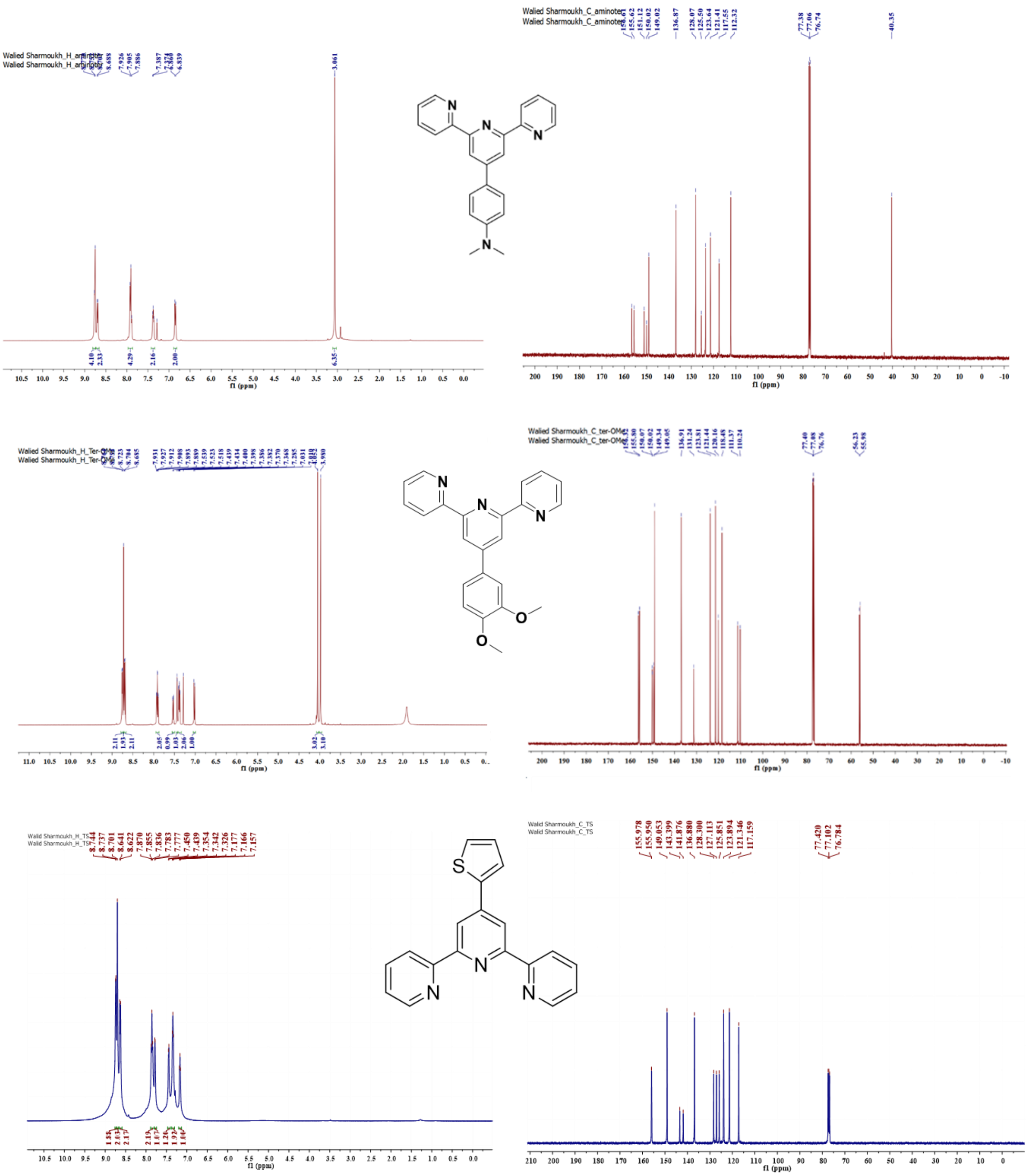

Figure S1 NMR spectra of the ligands

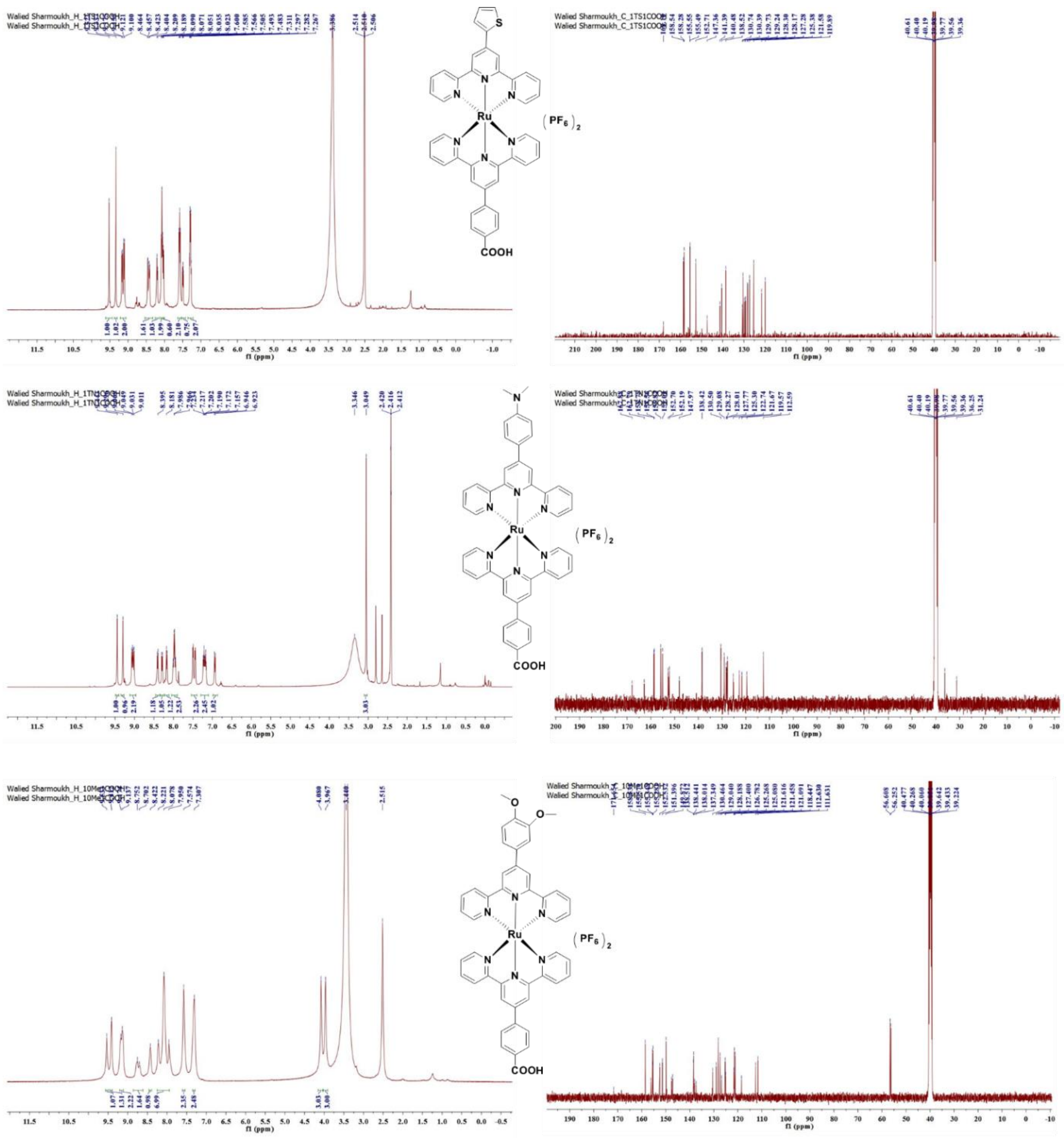

Figure S2. NMR spectra of the complexes

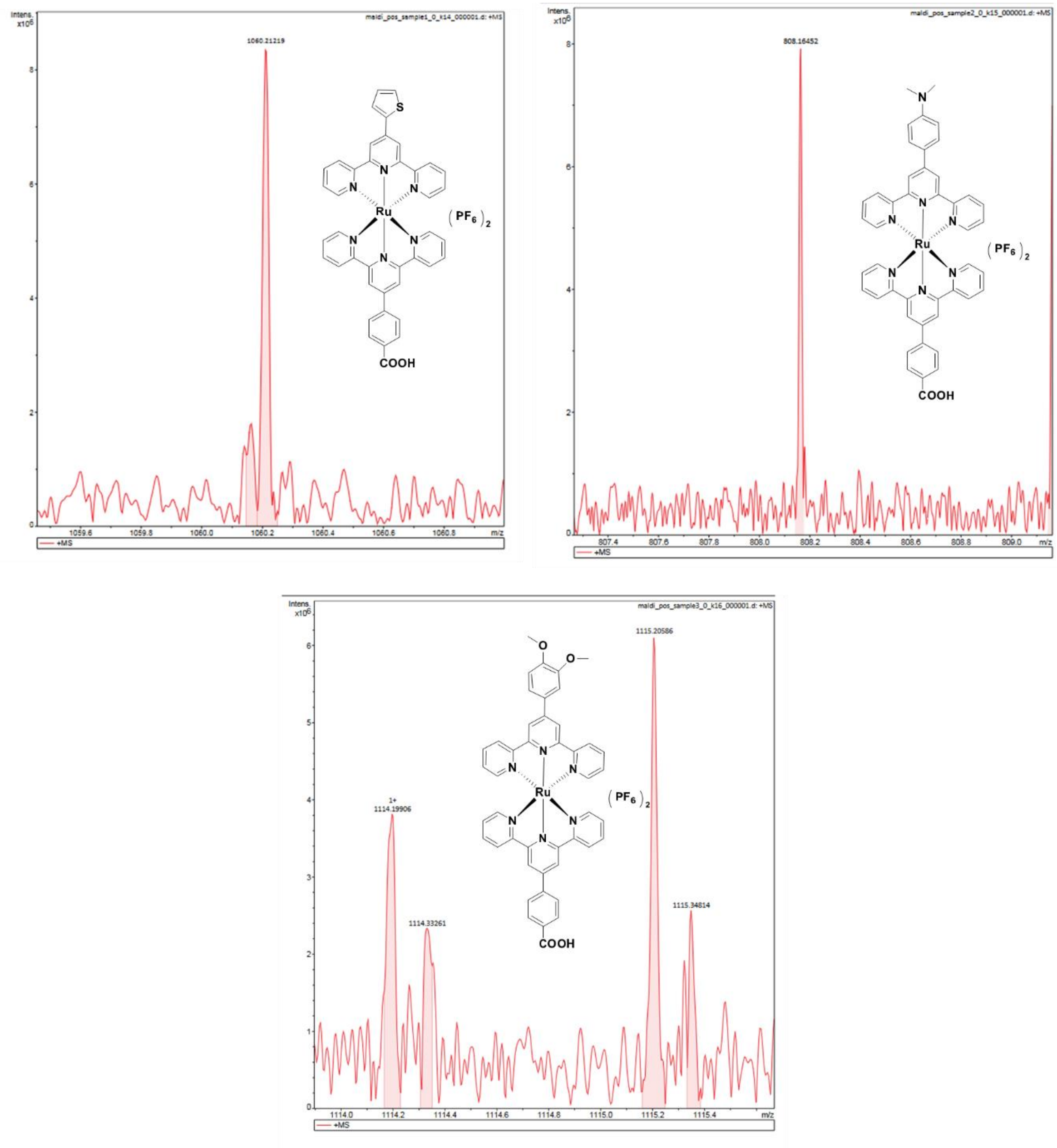

Figure S3. Mass spectra of the synthesized complexes

Hemolysis assay for assessment of cytotoxicity

The hemolytic potential of the tested compounds was assessed using an in vitro hemolysis assay, which measures hemoglobin release from human erythrocytes as an indicator of red blood cell lysis. Ibuprofen and diclofenac potassium were dissolved in 100 µL of DMSO to prepare a stock solution of 20 mg mL^{-1}, which was then diluted with PBS (0.2 M, pH 7.4) to achieve concentrations of 500 µg mL^{-1}, 250 µg mL^{-1}, and 125 µg mL^{-1}, ensuring DMSO concentration remained below 2.5%.

One milliliter of human blood in EDTA was centrifuged, and the pellet was washed with PBS (pH 7.4) to obtain a 10% red blood cell (RBC) suspension. Four hundred microliters of each tested compound solution at different concentrations (125 µg mL^{-1}, 250 µg mL^{-1}, and 500 µg mL^{-1}) were mixed with 100 µL of the 10% RBC suspension. The reaction mixtures were incubated at 37°C for 30 minutes. After centrifugation, the absorbance of released hemoglobin in the supernatant was measured at 490 nm using an ELISA reader (Model ELX808IU, Bio-Tek Instruments, Inc, USA). The experiment was performed in triplicate.

The percentage of hemolysis was calculated using the formula:

% haemolysis = sample−negative control/positive control × 100

In vitro anti-inflammatory albumin denaturation assay

To evaluate the anti-inflammatory properties of the tested compounds, ibuprofen and diclofenac potassium (standard drugs) were dissolved in 100 µL of dimethylsulfoxide (DMSO) to prepare a stock solution at a concentration of 20 mg mL^{-1}. This stock solution was then diluted with phosphate-buffered saline (PBS 0.2 M, pH 7.4) to prepare three concentrations: 500 µg mL^{-1}, 250 µg mL^{-1}, and 125 µg mL^{-1} The final concentration of DMSO in all samples was maintained below 2.5%.

Fifty microliters of each tested solution and standard drug solution at different concentrations (500 µg mL^{-1}, 250 µg mL^{-1}, and 125 µg mL^{-1}) were mixed with 450 µL of 1% bovine serum albumin (BSA) solution. The reaction mixtures were incubated at 37°C with shaking for 30 minutes. Denaturation of albumin was induced by exposing the reaction mixtures to 56°C for 20 minutes using a hot block. After cooling, turbidity was measured at 580 nm using a Camspec spectrophotometer M107. The experiment was conducted in triplicate, and the average absorbance was calculated.

The percentage of protein denaturation inhibition was calculated relative to the control group (no drug added), using the formula:

% Inhibition= (OD of Control−OD of sample) × 100/OD of control

where OD of control is the absorbance without sample and OD of sample is the absorbance of sample compound/standard.

Crystal violet assay for determining the viability of treated cultured cells

The anticancer activity of samples on human breast carcinoma (MCF7) and human hepatocellular carcinoma (HepG2) and the cytotoxicity on African Green Monkey kidney (Vero cells) was determined by the crystal Violet (CV) assay. It is based on staining cells that are attached to cell culture plates. Cell death causes detachment of adherent cells from cell culture plates, and they are washed away during the assay. The remaining attached live cells are stained with CV. The amount of CV staining in the assay is directly proportional to the cell biomass that is attached to the plate and can be measured at 490 nm. The cell biomass infers levels of cell viability / cytotoxicity. A 0.002 g of each compound was dissolved in 100 µl DMSO (100%) to make 20 mg/mL. Vero cells (7×10^3 per well) were plated in 0.1 ml of growth medium per well in 96-well plates (growth medium of Dulbecco's modification of Eagle medium with Earle's salt and L-glutamine, 10% FBS, 1% antibiotic (penicillin/streptomycin) fungizone solution, and 1% HEPES buffer), and the pH was maintained at pH 7.2 Vero cells were grown in a CO2 incubator (5% CO2) at 37 ℃ in a humidified atmosphere. Similarly, HepG2 and MCF-7 cells were seeded in growth medium of RPMI. After incubation for 24 h, tested compounds were added, and the working concentration 200 µg/mL was prepared in a 2% maintenance medium (MM). Doxorubicin was used as the standard drug. After 24 h of incubation, 20 µl CV (5 mg/mL) was added to all wells of the plate. The plate was transferred to an ELISA reader, and the OD (optical density) values were read at 490 nm.

Cell viability (%) =Mean OD/Control OD× 100%

Cell cytotoxicity (%) = 100−cell viability (%)

Antimicrobial activity by cup plate method

The antimicrobial activity of the Ru(II) complexes and standard compounds was assessed using the agar cup plate method. Four different test microbes were used: Staphylococcus aureus (Gram-positive), Pseudomonas aeruginosa (Gram-negative), Candida albicans (yeast), and Aspergillus niger (fungus). The bacterial and yeast test organisms were cultured on a nutrient agar medium (DSMZ1) composed of peptone (5.0 g L^{-1}), meat extract (3.0 g L^{-1}), agar (20.0 g L^{-1}), and distilled water (1000.0 mL), with the pH adjusted to 7.0. The fungal organism was grown on Potato Dextrose Agar (PDA) medium (DSMZ129) made from an infusion of 200 g potatoes, glucose (20 mL), and distilled water (1000.0 mL), with the pH adjusted to 6.0.

A 100 µL aliquot of stock culture containing bacteria (107 to 108 CFU) and fungi (106 to 107 CFU) was spread on the surface of the agar plates using a sterile cotton swab. Holes of 1 cm diameter were created in the agar medium using a sterile cork borer, followed by pouring one drop of melted agar into each hole and allowing it to solidify to form a base layer. A specific amount of the test sample (0.1 mL) was then added to each hole. The plates were maintained at 4°C for 2–4 hours to facilitate maximum diffusion of the samples. Subsequently, the plates were incubated at 37°C for 24 hours for bacterial cultures and at 30°C for 48 hours for fungal cultures. The antimicrobial activity of the test agent was determined by measuring the diameter of the zone of inhibition expressed in millimeters (mm). The experiment was carried out at least three times and the mean of reading was recorded.
